# Nonclassical dynamic modeling of nano/microparticles during nanomanipulation processes

**DOI:** 10.3762/bjnano.11.13

**Published:** 2020-01-13

**Authors:** Moharam Habibnejad Korayem, Ali Asghar Farid, Rouzbeh Nouhi Hefzabad

**Affiliations:** 1 Robotic Research Laboratory, Center of Excellence in Experimental Solid Mechanics and Dynamics, School of Mechanical Engineering, Iran University of Science and Technology, Narmak, Tehran, Iran; 2 Department of Mechanical Engineering, Science and Research Branch, Islamic Azad University, Tehran, Iran

**Keywords:** atomic force microscopy, modified couple stress theory, nanomanipulation, nanoparticle modeling, size effects

## Abstract

Since the manipulation of particles using atomic force microscopy is not observable in real-time, modeling the manipulation process is of notable importance, enabling us to investigate the dynamical behavior of nanoparticles. To model this process, previous studies employed classical continuum mechanics and molecular dynamics simulations which had certain limitations; the former does not consider size effects at the nanoscale while the latter is time consuming and faces computational restrictions. To optimize accuracy and computational costs, a new nonclassical modeling of the nanomanipulation process based on the modified couple stress theory is proposed that includes the size effects. To this end, after simulating the critical times and forces that are required for the onset of nanoparticle motion on the substrate, along with the dominant motion mode, the nonclassical theory of continuum mechanics and a developed von Mises yield criterion are employed to investigate the dynamical behavior of a cylindrical gold nanoparticle during manipulation. Timoshenko and Euler–Bernoulli beam theories based on the modified couple stress theory are used to model the dynamics of cylindrical gold nanoparticles while the finite element method is utilized to solve the governing equations of motion. The results show a difference of 90% between the classical and nonclassical models in predicting the maximum deflection before the beginning of the dominant mode and a difference of more than 25% in the dynamic modeling of a 200 nm manipulation of a gold nanoparticle with a length of 25 µm and aspect ratio of 30. This difference increases with each increment of the aspect ratio and reduction of manipulation distance. Furthermore, by applying an extended von Mises criterion on the modified couple stress theory, it is found that the failure aspect ratio of a cylindrical gold nanoparticle based on nonclassical models is 212% more than that of the classical model. In the end, the results are compared with those of the classical method on polystyrene nanorods. The results for cylindrical gold nanoparticles indicate that the material length scale has a major effect on the exact positioning of cylindrical nanoparticles.

## Introduction

It is not possible to simultaneously observe and manipulate a nanoparticle using atomic force microscopy (AFM) as the imaging and manipulation tools are combined. As a result, dynamic modeling and simulation are essential in this field of research. For the first time, Sitti and Hashimoto proposed a two-dimensional (2D) model for manipulation of spherical nanoparticles [1] based on which Tafazzoli and Sitti presented a model for describing the dynamic modes of nanoparticles during the manipulation process and obtained the associated critical forces and times [2]. Shen et al. studied the dynamical behavior of dagger cantilevers using the power series and employed the finite element method (FEM) to validate the outcomes. The classical Euler–Bernoulli beam theory was used for deriving the equations of motion. They also studied the sensitivity of cantilever frequencies to contact stiffness and investigated the variation of sensitivity with cantilever slope [3–4]. Shi and Zhao examined the contact models at the nanoscale and compared Derjaguin–Muller–Toporov (DMT), Johnson–Kendall–Roberts–Sperling (JKRS) and Maugis–Dugdale (MD) models with the Hertz model. They studied the effect of dimensionless load and the transition parameter on the contact area. They emphasized the importance of the MD model that covers a large area of AFM surveys [5].

Owing to the importance of the AFM cantilever spring constant and its use in calculation of the rupture force of protein bonds and Young’s modulus of nanoparticles, Clifford and Seah determined the AFM cantilever normal spring constant [6]. Korayem and Zakeri studied the effects of different parameters on the times and forces in a 2D manipulation. Using their proposed algorithm, the location of the nanoparticle up to the final position could be simulated [7]. Moradi et al. modeled the manipulation of cylindrical nanoparticles by means of AFM and a classical continuum mechanics approach. It was determined that there exists a difference between the dynamic mode of nano- and microbars. They found that the dominant dynamic mode for microrods and nanorods are rolling and sliding, respectively [8].

In a further development in modeling the manipulation process, Babahosseini et al. presented a 2D model by considering the influential parameters in nanoscale modeling. They employed the modified Coulomb and Lund–Grenoble (LuGre) theories for frictional models [9]. Hou et al. studied the behavior of cylindrical nanoparticle motion during the manipulation process. They considered the viscous friction and studied two states: turning the axis inside or outside of the nanoparticle [10].

Kahrobaiyan et al. investigated the resonance frequencies and sensitivity of the AFM cantilever using the modified couple stress theory (MCST). An analytical formulation was derived for natural frequencies by writing the differential equations of cantilever motion. They found that when the dimensionless thickness of beam is less than 10, the results of classical and nonclassical models are significantly different [11]. In another study, the nonlinear behavior of the cantilever came to attention [12].

In another use of MCST in manipulation process by means of AFM, Lee and Chang focused on the sensitivity of V-shaped cantilevers. The results showed that for a lower contact stiffness, the sensitivity of V-shaped cantilevers based on MCST is less than that based on classical theory. They concluded that stiffer cantilevers are suitable for scanning stiffer plates while softer cantilevers, which have a higher sensitivity, could be used for biological nanoparticles [13]. Using MSCT for modeling AFM with a piezoelectric system was considered in another study [14]. Polyakov et al. examined the dependence of static friction and contact area on nanoparticle geometry in the manipulation of spherical silver and polyhedral gold nanoparticles. Their employed models for the contact area of spherical nanoparticles were the frozen droplet model and DMT-M (the DMT model with Maugis’ approximation) [15]. Due to the vulnerability of the biological nanoparticles to the applied force, modeling the required manipulation forces is of considerable importance. Korayem and Saraee studied the effective forces in three-dimensional (3D) manipulation of biological nanoparticles for the first time. The simulation results were compared with those obtained from modeling the gold nanoparticle manipulation. In addition, the 3D stiffness matrix for a rectangular cantilever was presented for the first time [16].

Kawai et al. turned the spotlight on the superlubricity of graphene nanoparticles sliding on a gold substrate considering both computational and experimental approaches [17]. Liu et al. introduced a new strategy for manipulating nanoparticles. Mechanical modeling and finite element simulation were employed to analyze the behavior of samples and optimize the manipulation method. The model used for mechanical modeling was the classical Euler–Bernoulli beam model [18]. AFM cantilever dimensions are at micrometer scale and designers are constantly trying to make them smaller to achieve higher sensitivity and resolution. Jazi et al. tried to develop a more accurate model of the AFM cantilever using MCST by considering its size-dependent nature. They considered Euler–Bernoulli beam assumptions to model the dynamical behavior of cantilever. Studying the amplitude of the free vibrations of the cantilever showed the importance of including size effects in modeling. Also, stability analysis and frequency response of the microscope in the classical and nonclassical models were investigated. The results showed that considering size effects has a remarkable impact on a reliable estimation of the dynamical behavior of AFM [19].

Sharifi et al. simulated the interaction force between the AFM probe and surface. They used the force calculation capability of AFM and artificial neural network for simulation. The results showed that their proposed neural network was able to model the behavior of a probe in the noncontact model [20]. Yuan et al. focused on the problem of tip location uncertainty caused by the nonlinearity of piezoelectric and temperature changes. They proposed a method in which local scanning was used for observing the distance. The experimental results were consistent with their proposed algorithm [21]. Wu et al. studied the automated manipulation of flexible nanowires using AFM. Although the automated manipulation of solid nanoparticles was already investigated, it was not generalizable to flexible nanowires due to the complexity of flexible behavior. Also, for manipulating multiple nanowires, they presented a method based on graph theory that saved significant time owing to being independent from intermediate scanning [22].

Mahdjour Firouzi et al. tried to simulate the manipulation of biological nanoparticles using molecular dynamics. They used the single-walled carbon nanotubes as a probe and performed a series of simulations for studying the effects of various conditions on the success of the nanomanipulation process. They also studied two different strategies for protein manipulation [23]. In another study, using molecular dynamics simulation and a multiscale approach, Korayem et al. investigated geometrical effects on the manipulation of carbon allotropes [24].

Ghattan Kashani et al. presented a new method to overcome the adhesion force between the tip and nanoparticle while releasing the nanoparticle. They created the required repulsive force for releasing nanoparticles via high electrostatic voltage. The method was proposed for a conductive tip and nanoparticles, and the efficiency of the proposed method was studied using a combination of molecular dynamics simulation and FEM [25]. To examine the size dependence in the manipulation process by considering two fields on the nanometer and micrometer scale related to the nanoparticle and cantilever, respectively, Korayem et al. modeled the manipulation in vacuum, liquid and humid environments. Their proposed model contained a nonclassical model of MCST for the cantilever and tip and a molecular dynamics model for the nanoparticle. The results showed that the predicted changes in the nonclassical model are less than in the classical model [26].

The experimental studies indicate that size-dependent behavior plays a major role in nano/microstructures where the classical continuum mechanics are unable to predict this behavior. In the molecular dynamics method, in spite of accuracy, the dimensional problem arises, and due to hardware limitations, particles with more than a few hundred nanometers cannot be modeled. In this article, unlike in previous studies in which the modeling of nanoparticle dynamics has been performed by means of either classical theory of continuum mechanics or molecular dynamics simulation, the nonclassical theory of continuum mechanics is employed to study the dynamical behavior of cylindrical nanoparticles. In addition, a developed version of the von Mises criterion based on MCST is employed to evaluate the size-dependent yielding moments of the nanoparticle. The results are compared with those of classical models.

To this aim, the dynamic modeling of cylindrical nanoparticles studied in this paper is composed of four main steps. First, critical forces and times and the dominant motion mode of the cylindrical nanoparticle are modeled using kinematic and dynamic equations governing the probe and substrate of AFM. In this study, a 2D manipulation process is studied by considering the sliding and rolling modes of the nanoparticle. The critical time and force of the dominant motion mode are used as the inputs of next steps. After applying the exerted force on the nanoparticle by AFM and distributed resistant force resulting from friction and adhesion, deflections of the cylindrical nanoparticle before the onset of motion in the dominant mode are calculated using MCST. For this purpose, classical and nonclassical Euler–Bernoulli and Timoshenko beam theories are utilized. In the next step, in order to ensure that the cylindrical nanoparticle does not enter the failure zone, the existing stresses are studied using two classical and nonclassical yield criterion. The models employed in this analysis are the classical and nonclassical von Mises criterion. In the end, after calculating the nonclassical deflections of the cylindrical nanoparticle and ensuring that the sample will not fail under existing loading conditions, the motion of the cylindrical nanoparticle in the dominant mode is simulated and compared with the existing results of the classical model.

## Modeling and Theory

### Modeling critical forces and times and the dominant motion mode

The modeling of critical forces and times as well as the dominant motion mode of a cylindrical nanoparticle is done using kinematic and dynamic equations governing the problem and employing contact models appropriate for use at the nanometer scale. The employed approach has been used and verified in many studies [2,7,16]. The AFM used in this study is composed of a rectangular cantilever and a tip with a spherical contact point and the particle is manipulated on a silicon substrate. The Lundberg cylindrical contact model is chosen for the particle and substrate contact area, and the JKRS model is employed for particle–tip contact. Also, a modified Coulomb friction model is employed to represent the adhesion contact in particle and substrate contact area.

**Figure 1:**
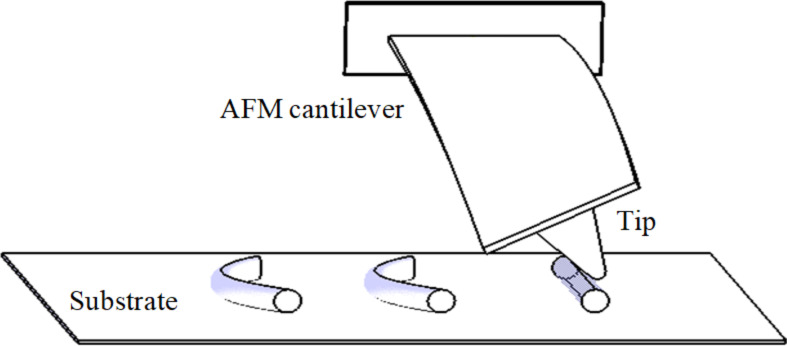
Schematic of the manipulation process of a cylindrical nanoparticle by means of AFM.

After the initial contact between the tip and nanoparticle is made, the AFM cantilever begins to deform, and by substrate motion, the particle moves with the substrate (in stick mode). This results in the increase of applied force from the tip in addition to bending and twisting deformation of the cantilever. As the applied force increases, cantilever deflection, particle–tip and particle–substrate indentations increase until the applied force exceeds the resistant force resulting from friction and adhesion and the particle moves relative to the substrate up to the target point. The schematic of the manipulation process of the cylindrical nanoparticle is shown in Figure 1.

The required conditions to initiate the sliding and rolling modes of a cylindrical nanoparticle on the substrate in the lateral direction in a 3D approach are, respectively, expressed by [16]

[1]
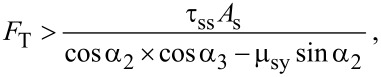


[2]
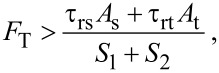


where


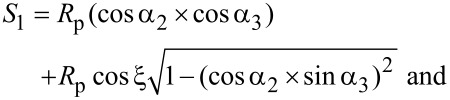



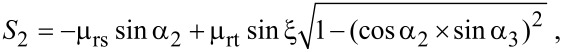


**Figure 2:**
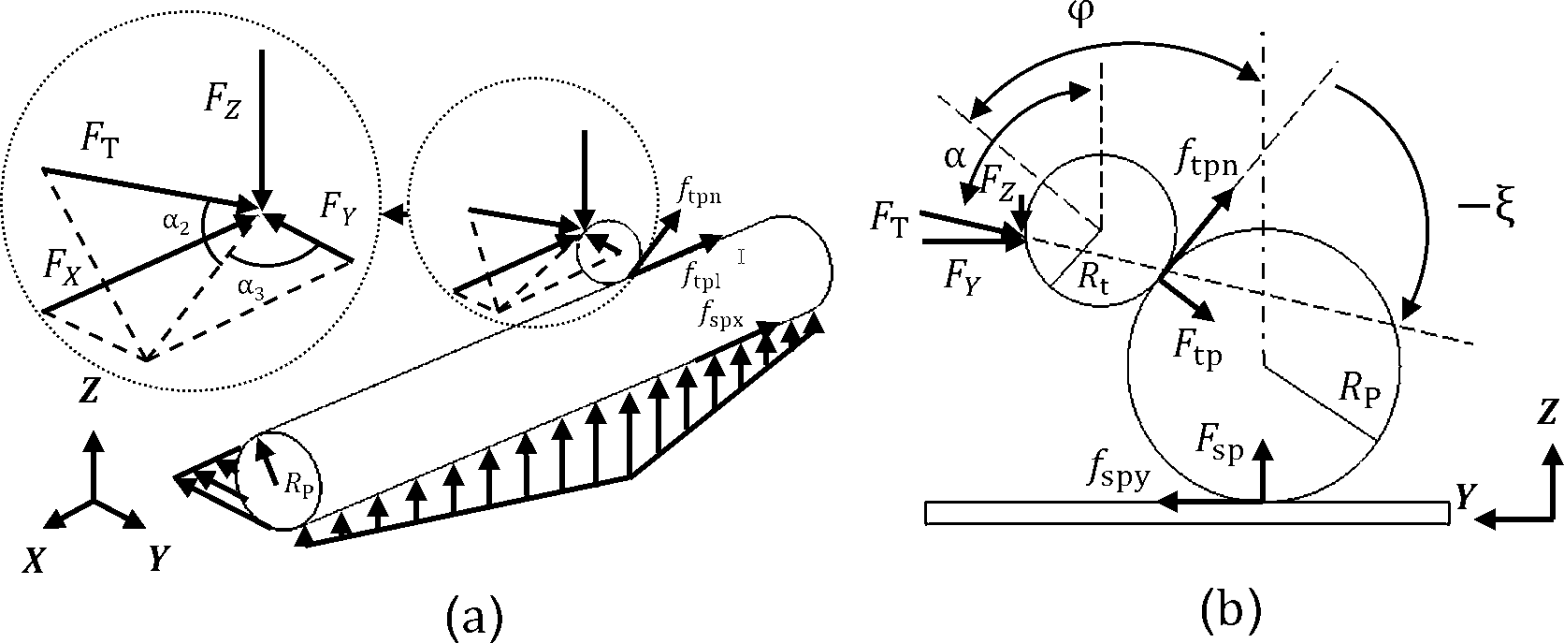
Nanoparticle manipulation forces and angles in a) 3D view and b) side view.

*F*_T_ is the pushing force, τ_ss_, τ_rs_ and τ_rt_ are the shear strength of contact in sliding on the substrate and rolling on the substrate and particle, respectively, μ represents the friction constants, *A*_s_ and *A*_t_ are the substrate–particle and particle–tip contact area, respectively, and *R*_p_ is the particle radius. The existing geometrical angles and exerted forces are shown in Figure 2. Since the established model in this paper is 2D involving the application of tip force to the middle of a cylindrical nanoparticle, critical forces and times and the dominant mode are obtained based on a 2D model.

### Governing equations using nonclassical theory

The considered models in this study are the Euler–Bernoulli and Timoshenko beam models based on MCST. The Strain energy according to MCST can be expressed as

[3]
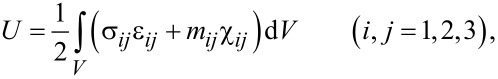


where σ_ij_, ε_ij_, are classical terms of stress and strain tensors, and *m*_ij_ and χ_ij_ are the symmetric part of couple stress and curvature tensors, respectively. Also, the relation for couple stress and curvature tensors is written as [27]

[4] $$m_{ij}=2l^{2}G\chi_{ij},$$

where *l* is the material length scale parameter for considering the size effects, and *G* is the shear modulus.

**Figure 3:**
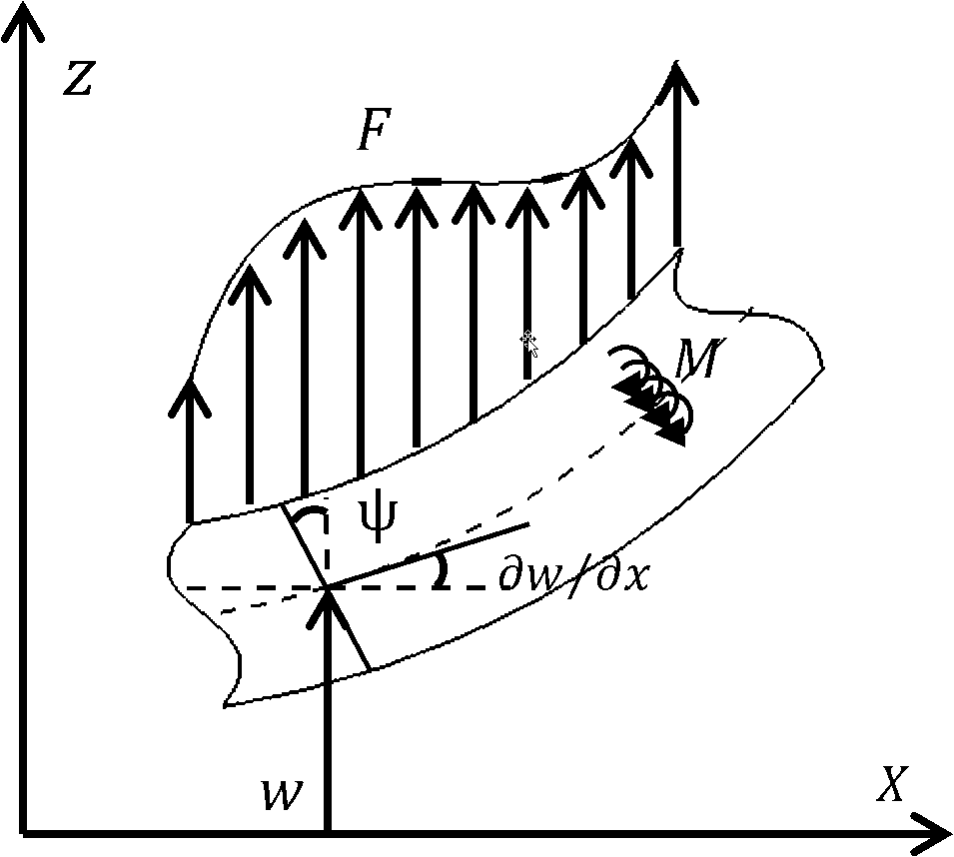
Timoshenko beam model: kinematic parameters, loading and coordinate system [27].

The displacement fields for the Timoshenko beam of Figure 3 are described as

[5]
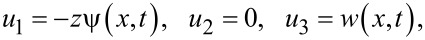


where *u*_1_, *u*_2_ and *u*_3_ are displacements along the axes *x*, *y* and *z*, respectively, ψ(*x*,*t*) is the angular rotation of the beam cross section and *w*(*x*,*t*) is the beam lateral deformation.

By replacing the equations of non-zero elements, one can obtain the strain, stress, curvature and couple stress matrices along with the non-zero elements of the rotation vector. Additionally, the strain and kinetic energy and the work of external force applied on the beam element are, respectively, defined as [27].

[6] $$U=\frac{1}{2}\int_{0}^{L}\left[\begin{array}{l} EI{\left(\frac{\partial \psi }{\partial x}\right)}^{2}+GA{\left(\frac{\partial w}{\partial x}-\psi \right)}^{2}\\ +\frac{Gl^{2}A}{4}{\left(\frac{\partial \psi }{\partial x}+\frac{\partial^{2}w}{\partial x^{2}}\right)}^{2} \end{array}\right]\text{d}x\;,$$

[7]
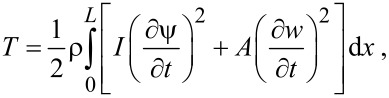


[8]
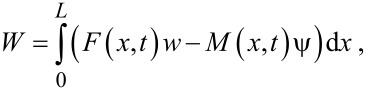


where *E* is the Young’s modulus, *L*, *A* and *I* are length, cross-sectional area and moment of inertia of the beam, respectively. ρ is the beam density and *F*(*x*,*t*) and *M*(*x*,*t*) designate the external force distribution and bending moment per unit length, respectively. By employing Hamilton’s principle, the equations of motion are obtained as [27]

[9]
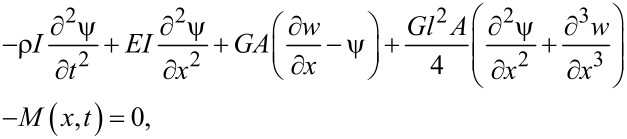


[10]
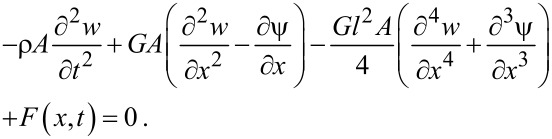


Using a trend similar to the presented approach for obtaining the equations of the Timoshenko beam, the equation of the Euler–Bernoulli beam based on the nonclassical MCST in static form is found to be [28]

[11]
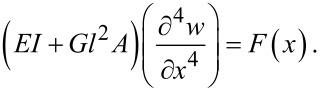


In order to derive mass and stiffness matrices for the beam element that consists of two nodes with two degrees of freedom, the nodal displacement vector *d*, displacement *w*(*x*,*t*), rotation ψ(*x*,*t*) and shape function matrices *N*^w^ and *N*^ψ^ must be determined. The procedure leads to the following stiffness and mass matrices and nodal force vector for the finite element solution [27]:

[12] $$K=\int_{L}^{0}\left\{\begin{array}{l} EI{\left(\frac{\partial N^{\psi }}{\partial x}\right)}^{T}\frac{\partial N^{\psi }}{\partial x}\\ +GA{\left(\frac{\partial N^{w}}{\partial x}-N^{\psi }\right)}^{T}\left(\frac{\partial N^{w}}{\partial x}-N^{\psi }\right)\\ +\frac{GAl^{2}}{4}{\left(\frac{\partial N^{\psi }}{\partial x}+\frac{\partial^{2}N^{w}}{\partial x^{2}}\right)}^{T}\left(\frac{\partial N^{\psi }}{\partial x}+\frac{\partial^{2}N^{w}}{\partial x^{2}}\right) \end{array}\right\}\text{d}x,$$

[13]
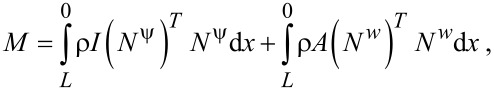


[14]
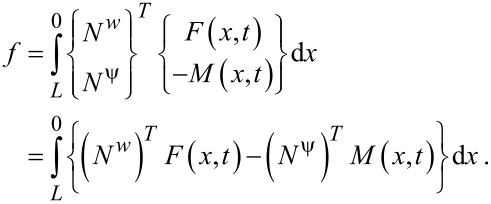


The governing equations can be expressed as

[15]
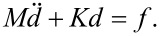


In addition, it is known that the behavior of the fracture mechanism at the nanometer and micrometer scale are quite different than at the macroscale [29]. Kahrobaiyan et al. [30] showed that an extension of von Mises criterion for a nonclassical Euler–Bernoulli beam based on MCST could be derived as

[16]
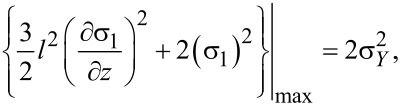


where σ*_Y_* is the yield stress and σ_1_ can be obtained from

[17]
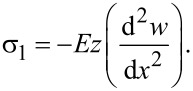


By considering the left-hand side of Equation 16 as the equivalent stress in the nonclassical model, the classical and nonclassical models could be compared by considering one yield stress as in

[18]
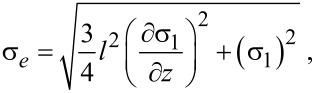


[19]
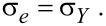


**Figure 4:**
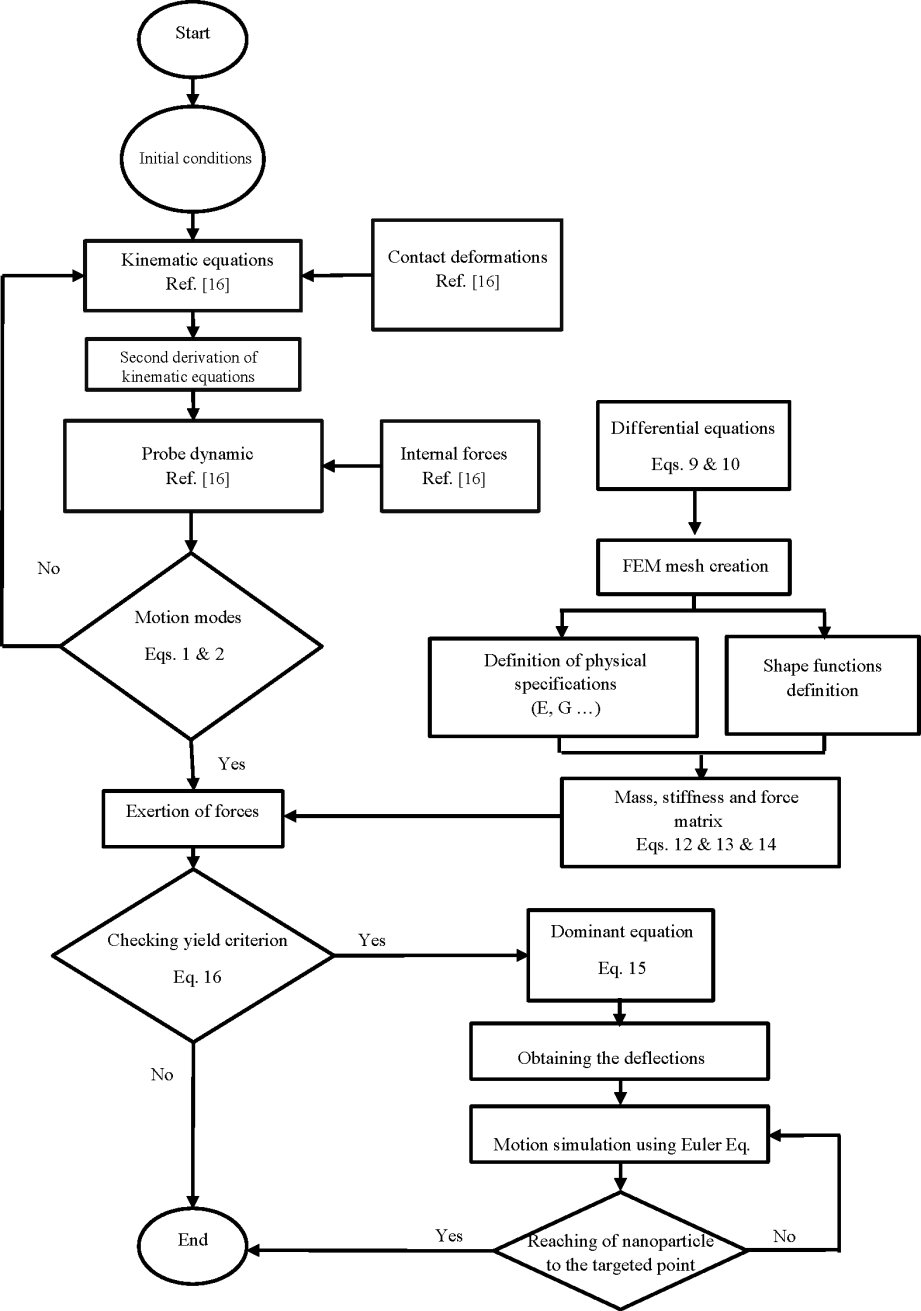
General algorithm for dynamic and mechanical modeling.

The general procedure for the dynamic modeling of the nanoparticle presented here is given by the algorithm of Figure 4.

## Results and Discussion

In this paper, the manipulation of a cylindrical gold nanoparticle by means of AFM with the properties listed in Table 1 on a silicone substrate with an energy level of ω = 0.2 j/m^2^ and tribological parameters according to Table 2 are studied.

**Table 1:** AFM properties of the cylindrical gold nanoparticle modeled in this study [7].

*L* (µm)	*w* (µm)	*t* (µm)	*H* (µm)	*R*_t_ (nm)	*E* (GPa)	ν	ρ (kg/m^3^)

225	48	1	12	20	2330	0.27	169

**Table 2:** Tribological parameters between particle/tip and needle/substrate [6].

µ_s_	µ_d_	µ_r_ (nm)	τ (MPa)	τ_r_ (Pa·m)

0.8	0.7	80	28	28

In Table 1 *L*, *w*, *t* and *H* represent length, width, thickness of cantilever and probe height respectively and in Table 2 µ_s_, µ_d_, µ_r_, τ and τ_r_ are the static, dynamic and rolling friction coefficients.

**Figure 5:**
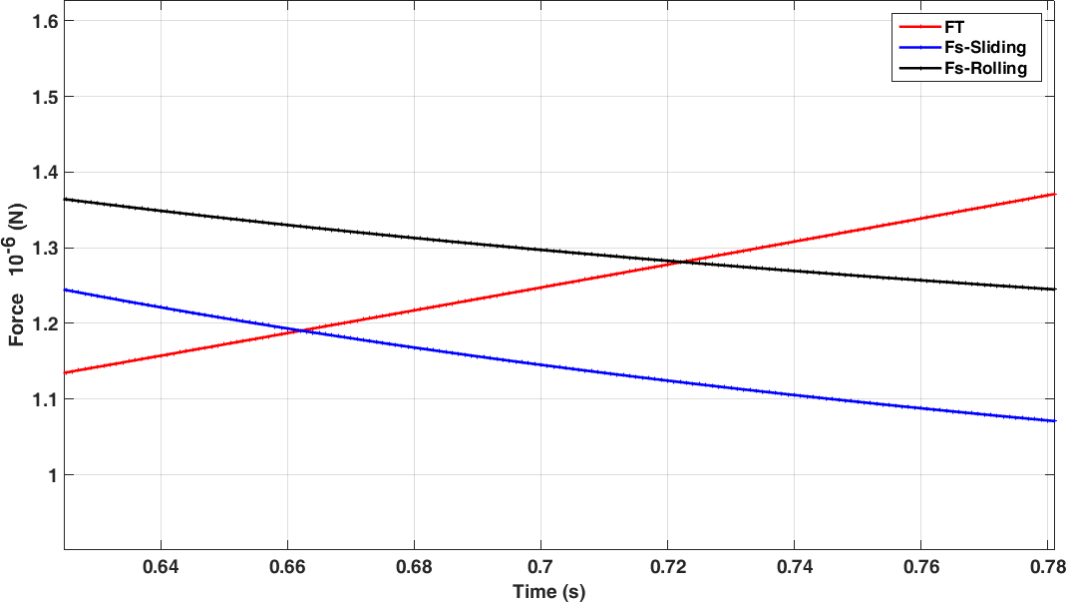
Simulation of critical times and forces in sliding and rolling modes for a gold nanoparticle with a length of 25 μm and aspect ratio of 30.

By considering Equation 1 and Equation 2 in the form *F*_T_ > *F*_s_, the critical forces and times at which the tip pushing force overcomes the resistance force are illustrated in Figure 5 for a cylindrical gold nanoparticle with a length of 25 µm and aspect ratio of 30 in rolling and sliding modes.

**Figure 6:**
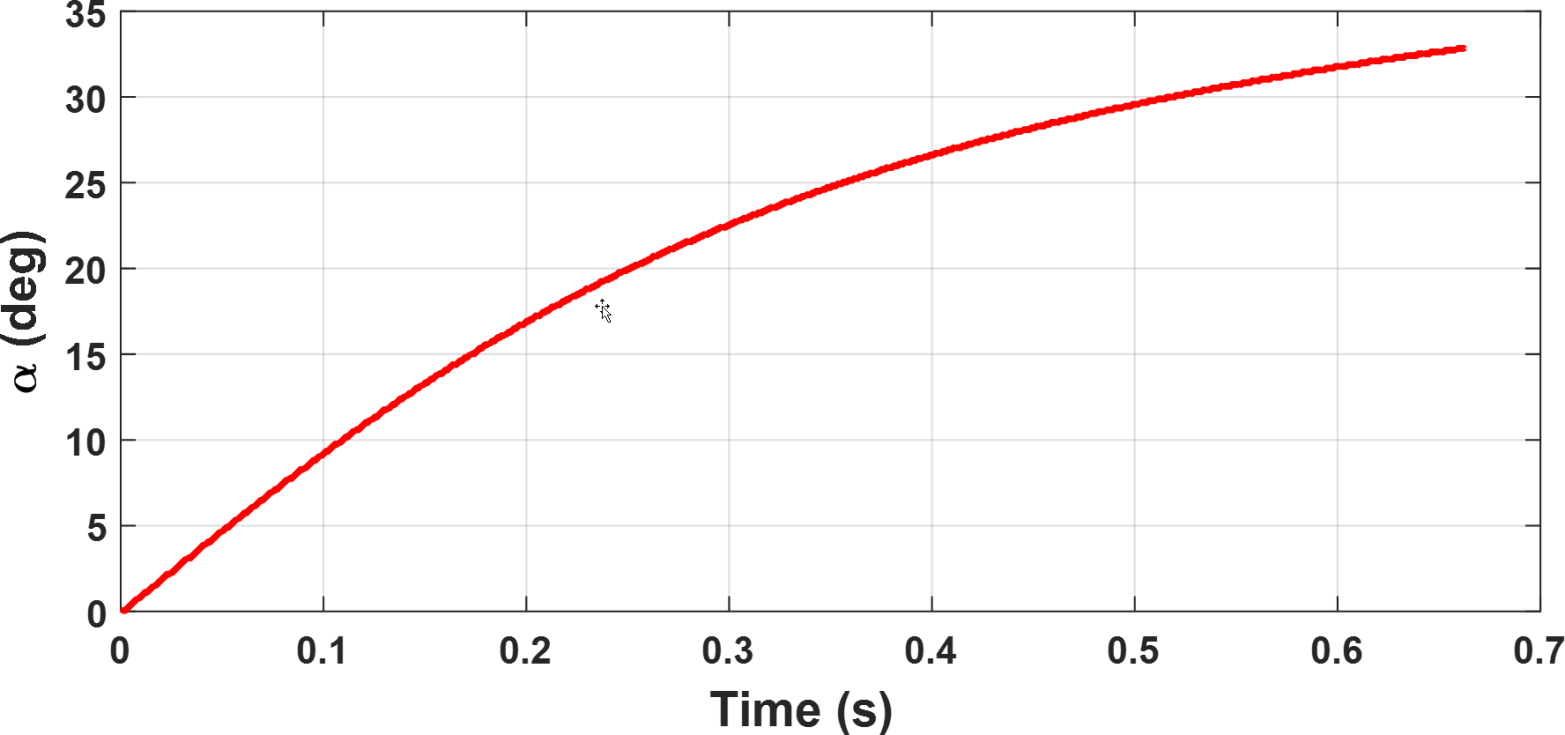
Simulations of the variation of the tip force angle (α) during the manipulation process until achieving the critical time for the sliding mode.

As observed, under the given conditions, the sliding mode is the dominant motion mode of a nanoparticle on the substrate, i.e., the sliding mode would start sooner with a lower amount of force than the rolling mode. By reducing the aspect ratio, the sliding mode transfers to rolling mode. For the case presented in this paper, aspect ratios of less than 15 for the gold nanoparticle could be considered in the rolling region and since the speed of the substrate is constant, the change in motion mode does not occur during the manipulation process. In the next sections, the critical time and force of the dominant mode are simulated and used according to the reviewed geometry. After achieving critical time and force, since the applied force along the direction of nanoparticle motion is required to study the motion dynamics, the tip force angle should be obtained. Figure 6 shows the variation of angle α (Figure 2b) during the manipulation process until achieving the critical time for the sliding mode. As can be seen, α starts from an angle of 0°, then increases until reaching the critical point at 32.84°. As stated regarding the use of critical force and time for different geometries, the new critical angle should be achieved. Modeling based on the nonclassical theory of MCST requires a material length scale parameter. This constant for the gold particle according to Fathalilou et al. [31] is considered to be *l* = 1.12 µm.

The design parameters in modeling a gold nanoparticle are presented in Table 3.

**Table 3:** Gold nanoparticle design parameters.

Parameter	Parameter value

*E* (GPa)	79
υ	0.44
ρ (kg/m^3^)	19300

**Figure 7:**
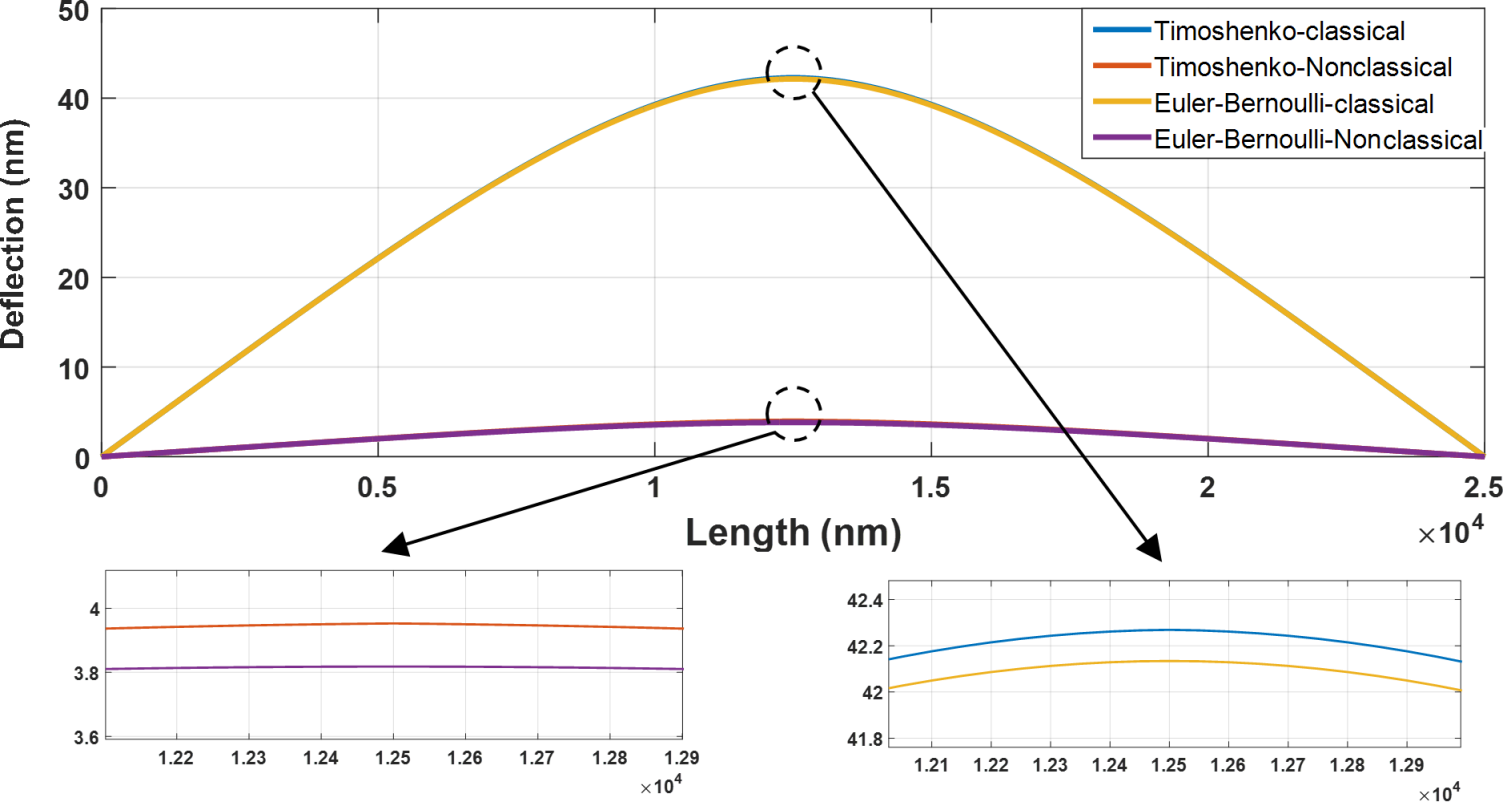
Classical and nonclassical modeling of the deflections of a cylindrical gold nanoparticle (length = 25 µm and aspect ratio = 30) based on the four models of classical Euler–Bernoulli, classical Timoshenko, nonclassical Euler–Bernoulli and nonclassical Timoshenko.

Since the cylindrical gold nanoparticle is flexible, the exerted forces lead to the deflection of nanoparticle prior to the onset of motion. Figure 7 shows the cylindrical gold nanoparticle deformation (length = 25 µm and aspect ratio = 30) based on the four models of classical Euler–Bernoulli, classical Timoshenko, nonclassical Euler–Bernoulli and nonclassical Timoshenko. By adding the material length scale parameter to classical equations, the deflections in nonclassical models are decreased. The calculated values of 42.27 and 42.13 nm in the classical Timoshenko and Euler–Bernoulli models are reduced to 3.95 and 3.82 nm in the corresponding nonclassical models.

According to Figure 7, a difference of more than 90% between the classical and nonclassical models demonstrates the importance of classical models in the analysis of nano/micrometer scale effects. Due to the large aspect ratio, the results of the nonclassical Timoshenko and Euler–Bernoulli models approach each other similar to classical models. It is noticeable that the results of nonclassical beams resemble the rigid body behavior but it should be taken into the account that this behavior is not the case with all material and dimensional characteristics. It will be later observed in the section “Comparison of the results with other studies” that for a polystyrene nanorod that is softer than gold the same conclusion can not be reached. Table 4 shows the maximum deflections for the analyzed sample.

**Table 4:** Comparison of the maximum deflection of a cylindrical gold nanoparticle with the length of 25 µm and aspect ratio of 30.

Beam model	Maximum deflection (nm)	Difference from classical Timoshenko

classical Timoshenko	42.27	0%
classical Euler–Bernoulli	42.13	0.33%
nonclassical Timoshenko	3.95	90.65%
nonclassical Euler–Bernoulli	3.82	90.96

**Figure 8:**
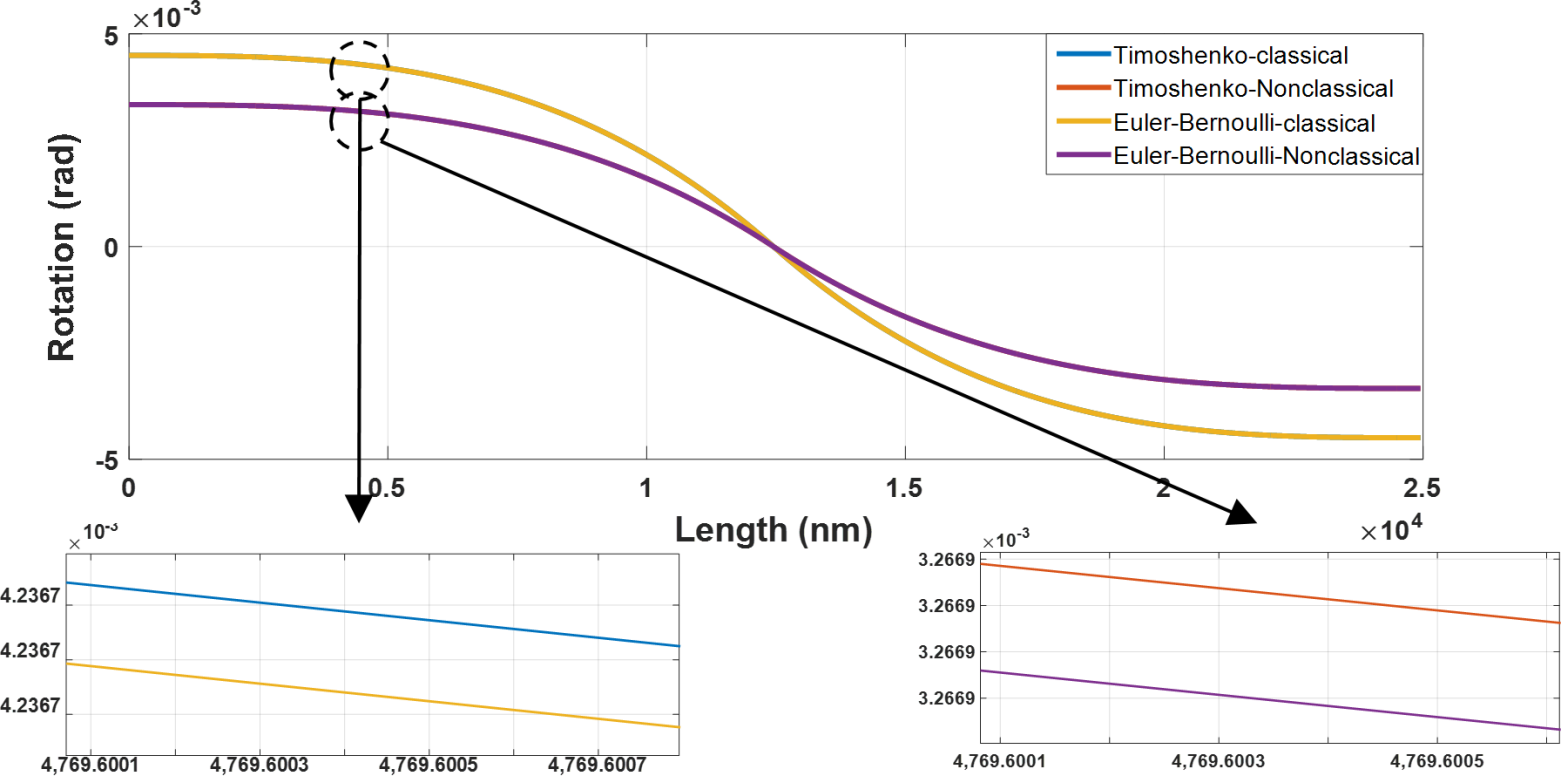
Classical and nonclassical modeling of the rotation angles of a cylindrical gold nanoparticle (length = 25 µm and aspect ratio = 30) based on the four models of classical Euler–Bernoulli, classical Timoshenko, nonclassical Euler–Bernoulli and nonclassical Timoshenko.

The rotation angle of the modeled cylindrical gold nanoparticle along the nanoparticle length for the four beam models is presented in Figure 8.

**Figure 9:**
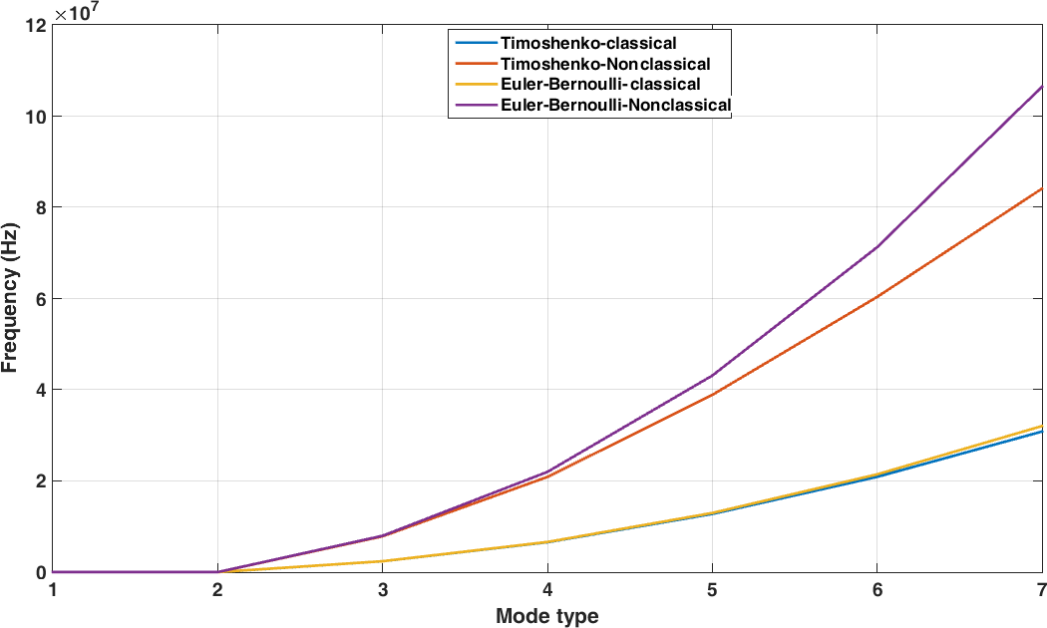
Natural frequencies of the classical and nonclassical models of a cylindrical gold nanoparticle with length of 25 μm and aspect ratio of 30.

The first seven natural frequencies of the studied sample are presented in Figure 9. In addition, Table 5 presents the dimensionless natural frequencies according to Figure 9. As observed, the natural frequencies in the classical and nonclassical Timoshenko models are less than in the corresponding Euler–Bernoulli models. The zero value of the first two frequencies of all models is a sign of rigid-body motion as two degrees of freedom are assigned to each node.

**Table 5:** Dimensionless natural frequencies of the cylindrical gold nanoparticle with length of 25 μm and aspect ratio of 30.

Mode	Euler–Bernoulli		Timoshenko	
	Nonclassical	Classical	Nonclassical	Classical

3	74.32	22.37	73.21	22.31
4	204.90	61.67	194.60	61.16
5	401.60	120.90	362.20	118.98
6	663.90	199.36	562.30	194.71
7	991.80	298.56	783.60	287.34

Regardless of the first two rigid modes (zero frequencies), the values of natural frequencies in comparison with the tip force duration until the critical time is reached indicates that the static mode is dominant.

### Sensitivity of deflections to the change in aspect ratio

**Figure 10:**
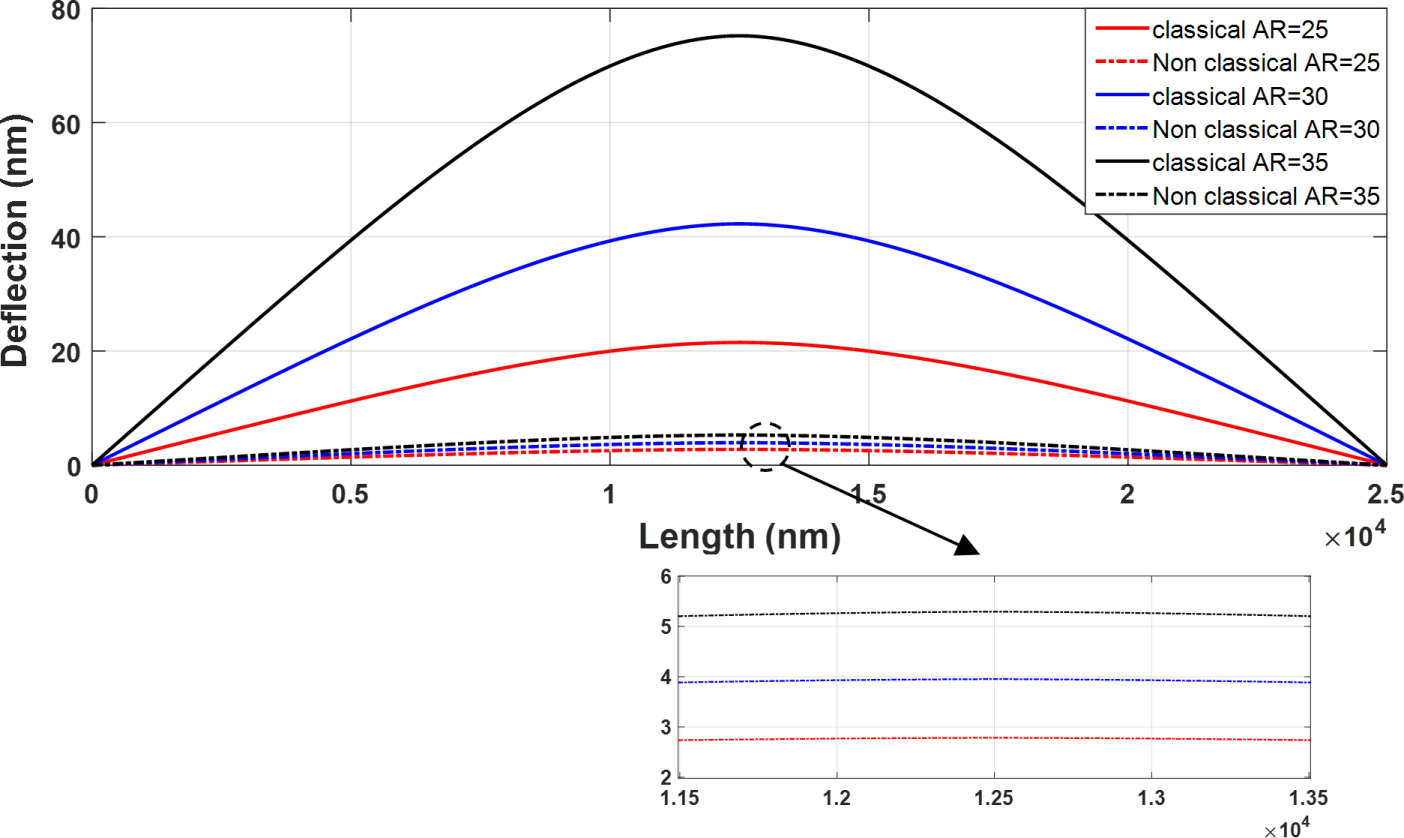
Sensitivity of classical and nonclassical Timoshenko beam model deflections to the change in aspect ratio.

To study the effect of aspect ratio on deflection, by keeping the length fixed and changing the particle radius, the results are presented in Figure 10 for three aspect ratios of 25, 30 and 35 based on the classical and nonclassical Timoshenko beam models.

The deflections in both classical and nonclassical models decrease with decreasing aspect ratio. For the aspect ratios of 35, 30 and 25, the maximum deflections of 75.2, 42.27 and 21.51 nm in the classical Timoshenko model reach 5.29, 3.95 and 2.87 nm in the nonclassical model, respectively. In addition, with decreasing aspect ratio, the difference between the classical and nonclassical models is reduced and classical and nonclassical models approach each other. It can be seen that the difference between classical and nonclassical models has decreased from 92.96% for the aspect ratio of 35 to 90.65% and 86.63% for the aspect ratios of 30 and 25, respectively. Thus, it is concluded that with a reduction in aspect ratio, the classical and nonclassical models tend to yield similar results and the impact of size effects decrease.

### Dynamic simulation using nonclassical models

**Figure 11:**
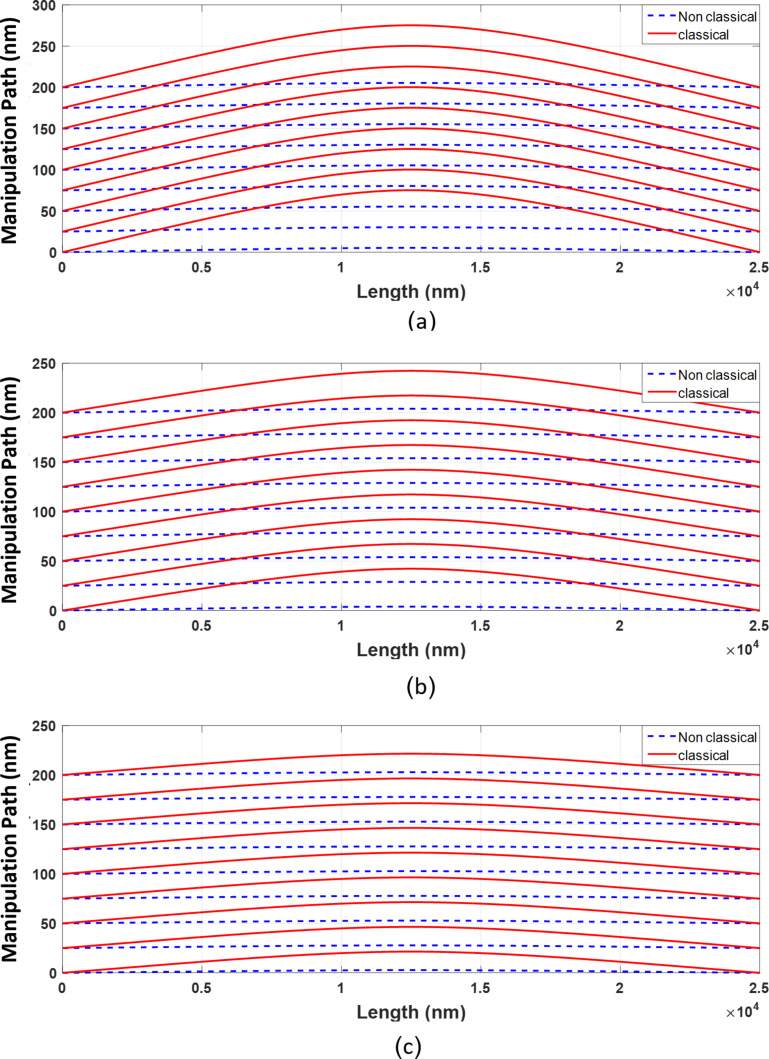
Simulation of motion of a cylindrical gold nanoparticle during a manipulation of 200 nm. A) Aspect ratio of 35. B) Aspect ratio of 30. C) Aspect ratio of 25.

Once the cylindrical nanoparticle reaches the critical time and force in the dominant mode (sliding), the maximum possible deflection before the onset of motion is obtained and the nanoparticle starts to slide on the substrate. But since the dynamic and static friction coefficient are different, the modified deflection of beam due to the dynamic coefficient must be employed to explain the nanoparticle behavior in motion mode. Due to this transition, the resistance force (and thus tip force) and the deflection of the particle reduce so that for the aspect ratio of 30 the tip force reduces by 6.58% (from 1.1903 × 10^−6^ N to 1.1120 × 10^−6^ N). With respect to deflections the maximum deflections of 42.27, 42.13, 3.92 and 3.82 nm for classical and nonclassical beam theories (Table 4) reduce to 39.49, 39.49, 3.69 and 3.56 nm, respectively. Now, with the purpose of studying the difference between classical and nonclassical models regarding the dynamic modeling of manipulation, the cylindrical gold nanoparticle in sliding mode is manipulated by a value of 200 nm at the substrate speed of 50 nm/s. Figure 11 and Table 6 show the final position of the cylindrical nanoparticle with a length of 25 µm and aspect ratios of 35, 30 and 25 according to the classical and nonclassical Timoshenko beam models.

**Table 6:** Final position of the cylindrical gold nanoparticle in a 200 nm manipulation (length = 25 µm and aspect ratio = 35, 30 and 25) according to the classical and nonclassical Timoshenko beam models).

Aspect ratio	25	30	35

final position in classical model	220.15	239.49	269.81
final position in nonclassical model	202.61	203.69	204.92
difference	7.97%	14.95%	24.05%

According to the results, by increasing the aspect ratio of the cylindrical nanoparticle, the difference between the classical and nonclassical models in predicting the final setting of nanoparticle increases. For the cylindrical gold nanoparticle with an aspect ratio of 35, an error of more than 24% (equivalent to 64.89 nm) is observed. By decreasing the aspect ratio, although the deflections of the two models decrease, the dominant mode turns from sliding to rolling. By considering the simulated critical times in Table 7, the required times for approaching the final point in classical models are obtained as 4.7030, 4.6620 and 4.6310 seconds for the aspect ratios of 25, 30 and 35, respectively. In contrast, the corresponding values for nonclassical models are 5.075, 5.4284 and 6.029 seconds, respectively.

**Table 7:** Critical forces and times of a cylindrical gold nanoparticle in sliding mode with respect to change in aspect ratio.

Aspect ratio	25	30	35

critical force (µN)	1.2520	1.1903	1.1440
critical time (s)	0.7030	0.6620	0.6310

By increasing the manipulation distance, the effects of applying the classical models are reduced. The final position after a 400 nm manipulation of the gold particle with aspect ratio of 35 is predicted to be 496.81 nm, whereas the classical model predicts a 404.92 nm manipulation. Therefore, it could be concluded that since the difference of 24.05% in the 200 nm manipulation is reduced to 18.49% from that of a 400 nm manipulation, by decreasing the manipulation distance, the classical model can be better employed. To gain a better understanding, it can be claimed that by decreasing the aspect ratio and increasing the manipulation distance, the results of classical and nonclassical models approach each other, and using classical models generates fewer errors. On the contrary, by increasing the aspect ratio and reducing the manipulation distance, the error associated with classical models rises.

### The sensitivity of manipulation dynamics to size-dependent effects

**Figure 12:**
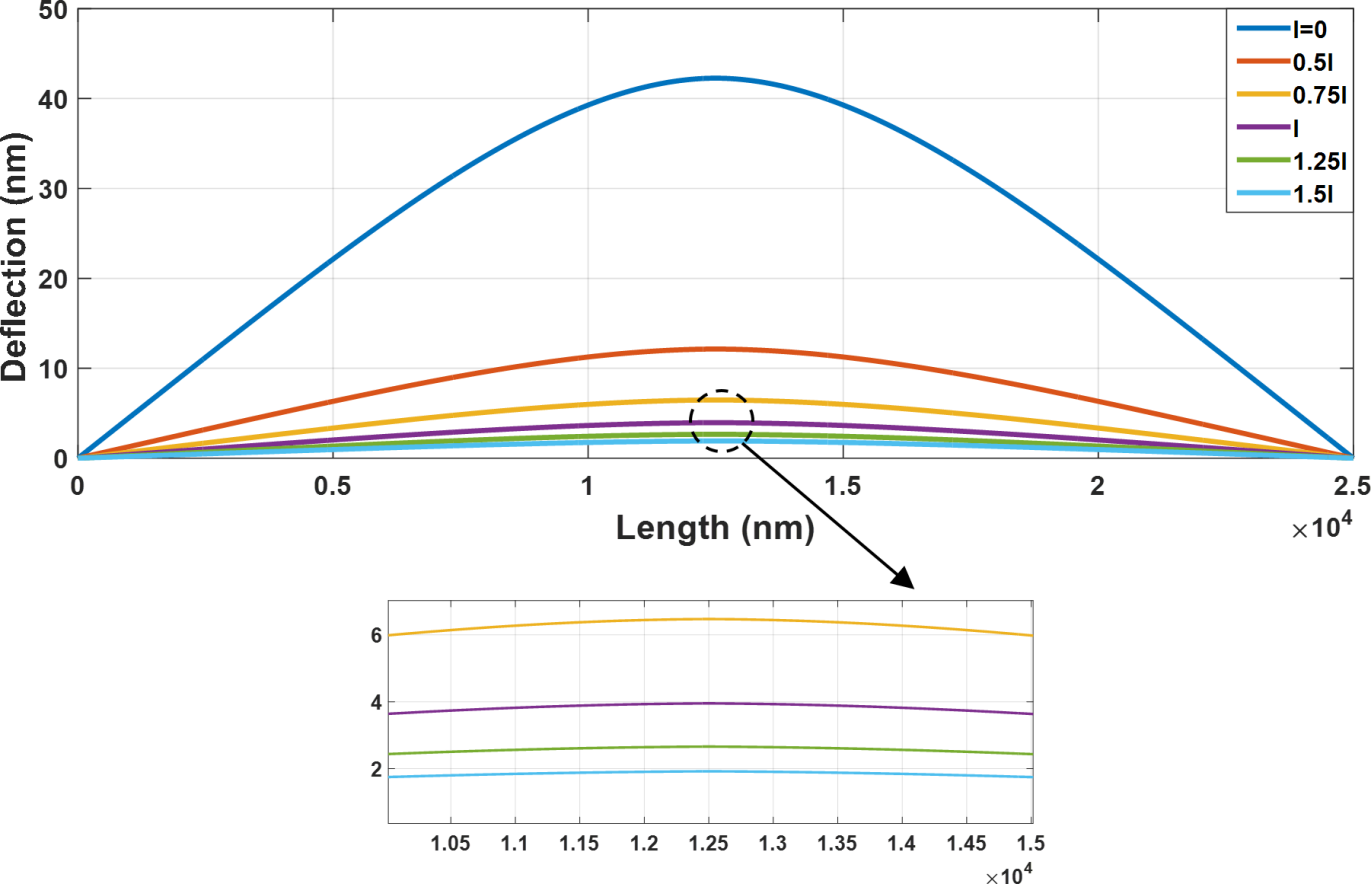
The sensitivity of manipulation dynamics to size effects (length = 25 µm and aspect ratio = 30).

Obtaining the material length scale parameter is realized via experimental methods, and its precise calculation has a great impact on the accuracy of related nonclassical models. In this section, by considering a value of 1.12 µm, the effects of the change in the material length scale parameter on the manipulation dynamics are investigated. For this reason, with the assumption of *l* changing from 0.5*l* to 1.5*l*, the maximum deflections in the classical (*l* = 0) and nonclassical Timoshenko models are shown in Table 8 and Figure 12.

**Table 8:** The sensitivity of dynamic of manipulation to size effects (length = 25 µm and aspect ratio = 30).

Material length scale parameter	1.5*l*	1.25*l*	*l*	0.75*l*	0.5*l*	*l* = 0

maximum deflection (nm)	1.92	2.66	3.95	6.48	12.14	42.27

**Figure 13:**
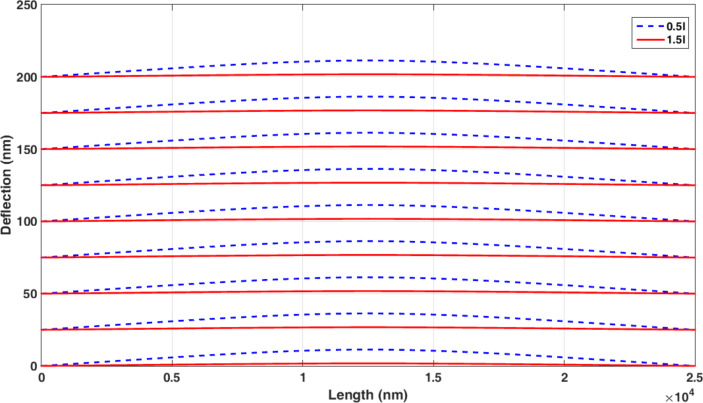
Manipulation of the nonclassical Timoshenko model for 0.5*l* and 1.5*l*.

The data of Table 8 indicate the importance of the exact determination of the material length scale parameter. It is observed that by increasing the parameter *l* from 0.5*l* to 1.5*l*, the predicted maximum deflection is decreased by 84%. Figure 13 shows the difference between the manipulation of the nonclassical Timoshenko model for the size coefficients of 0.5*l* and 1.5*l*.

**Figure 14:**
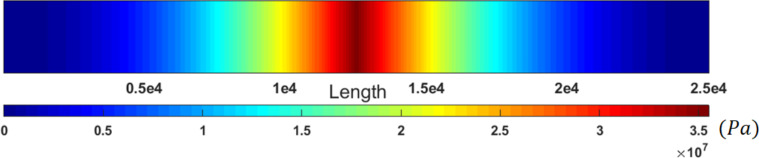
Classical stresses in a cylindrical gold nanoparticle with a length of 25 µm and aspect ratio of 30.

**Figure 15:**
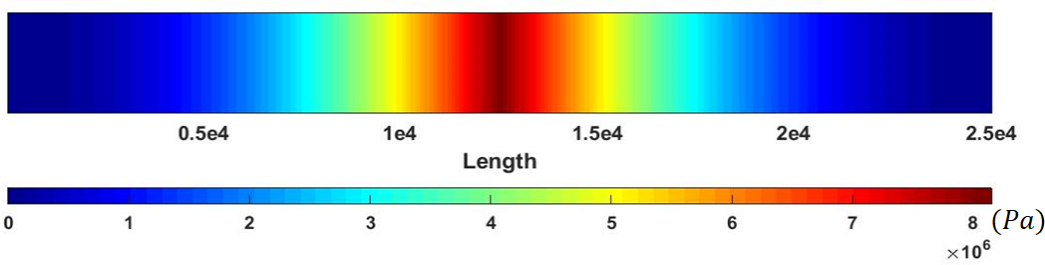
Equivalent stresses (nonclassical) in a cylindrical gold nanoparticle with a length of 25 µm and aspect ratio of 30.

In order to ensure that the sample will not fail, the stresses based on the classical Euler–Bernoulli beam and equivalent stresses in nonclassical counterpart are illustrated using FEM in Figure 14 and Figure 15, respectively. The maximum stress of 35.05 MPa in the classical model and maximum equivalent stress of 8.15 MPa in comparison with the yield stress of 200 MPa for gold [32] provide the required assurance.

**Figure 16:**
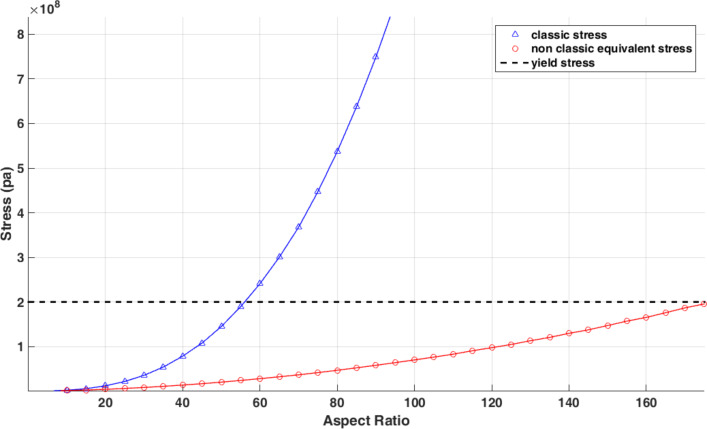
Comparison of the classical stresses and nonclassical equivalent stresses with respect to changes in aspect ratio.

Figure 16 shows the changes in classical stress and nonclassical equivalent stress of the Euler–Bernoulli beam with respect to the aspect ratio. It should be mentioned that for each aspect ratio, the exerted force should be calculated separately.

**Figure 17:**
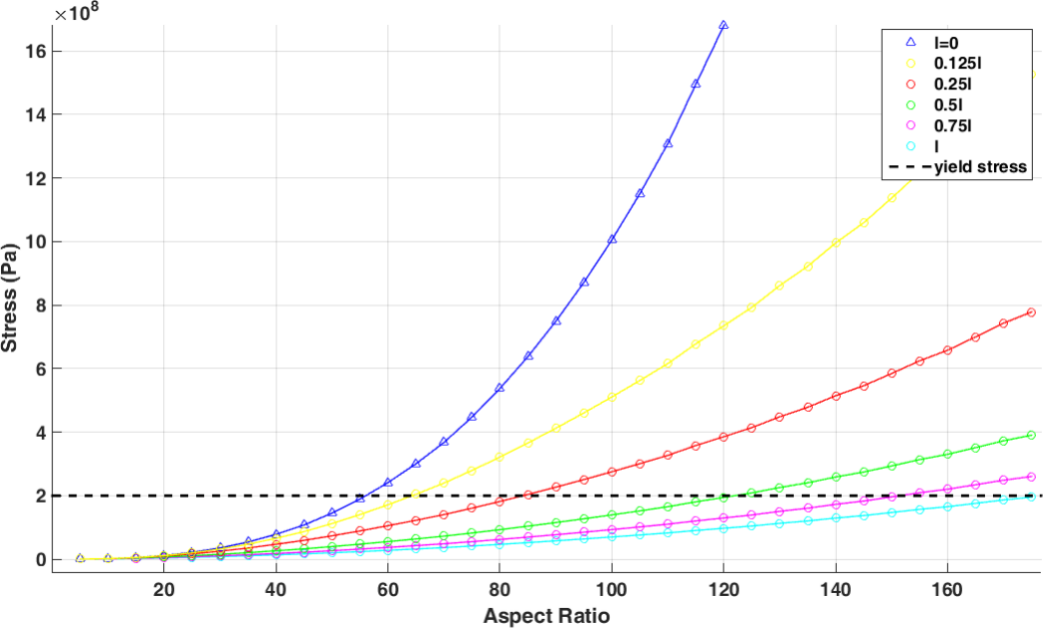
Variation of nonclassical equivalent stresses with material length scale parameter and change in aspect ratio.

Comparing the aspect ratio of 56 in classical theory with 175 in nonclassical theory reveals the importance of considering nonclassical models in predicting sample failure. Figure 17 shows the variation of nonclassical equivalent stress with the material length scale parameter. It is seen that by reducing the material length scale parameter, the nonclassical model approaches the classical model. Table 9 shows the aspect ratio of failure with respect to the material length scale parameter.

**Table 9:** Variation of the failure aspect ratio with the material length scale parameter.

Material length scale parameter	*l*	0.75*l*	0.5*l*	0.25*l*	0.125*l*	*l* = 0

failure aspect ratio	175	155	122	85	64	56

As observed in the section “Dynamic simulation using nonclassical model” where the critical forces and times are obtained, a dynamic simulation is performed using a contact and friction model appropriate for the nanometer scale. It can be seen that the obtained critical forces and times are the same in terms of classical and nonclassical models and employing the beam models is where the effects of nonclassical models emerge. Nonclassical theories of continuum mechanics include material length scale parameters in governing equations to capture the size effects in nanometer and micrometer scales. The nonclassical theory of modified couple stress employed in this paper includes one material length scale parameter (*l*) that contributes to the beam model established based on this nonclassical theory. As observed in Equation 13 for the nonclassical Euler–Bernoulli model, the term µ*AL*^2^ has been added to *EI* unlike the classical equation. It is obvious that by increasing the material length scale parameter, the value of equivalent stiffness as well as the discrepancy between classical and nonclassical models increases. By using the described analysis used for the Euler–Bernoulli beam model, similar results regarding the decrease of deflections in the Timoshenko beam model are attainable. The difference between Euler–Bernoulli and Timoshenko beam models are due to the additional degree of freedom of the Timoshenko beam model in both the classical and nonclassical approach. However, the difference vanishes when the sample under study is thin enough. The sensitivity of the models to the material length scale parameter and beam dimensional characteristics are widely discussed in the related article.

To address the behavior of the particle after retracting the tip, it should be mentioned that it depends on the beam dimensions and material characteristics along with the applied force. For this reason, the potential energy of the beam and the adhesion energy must be obtained, and if the potential energy of the beam is more than the adhesion energy the beam tends to retain its straight position [8].

### Comparison of the results with other studies

**Figure 18:**
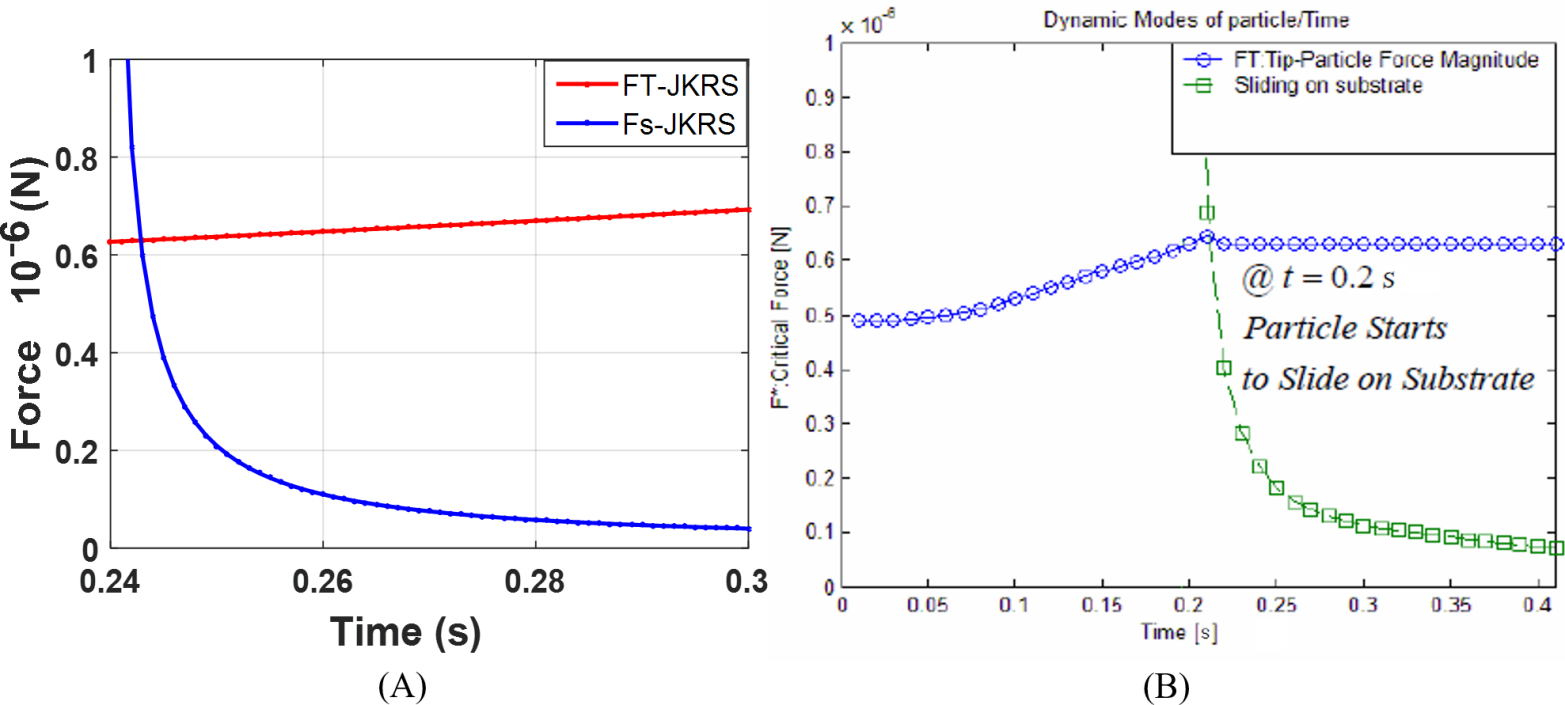
Comparison of the critical force and time simulation using the JKR contact model for a gold particle. A) Present study simulation method. B) Tafazzoli and Sitti simulation [2].

**Figure 19:**
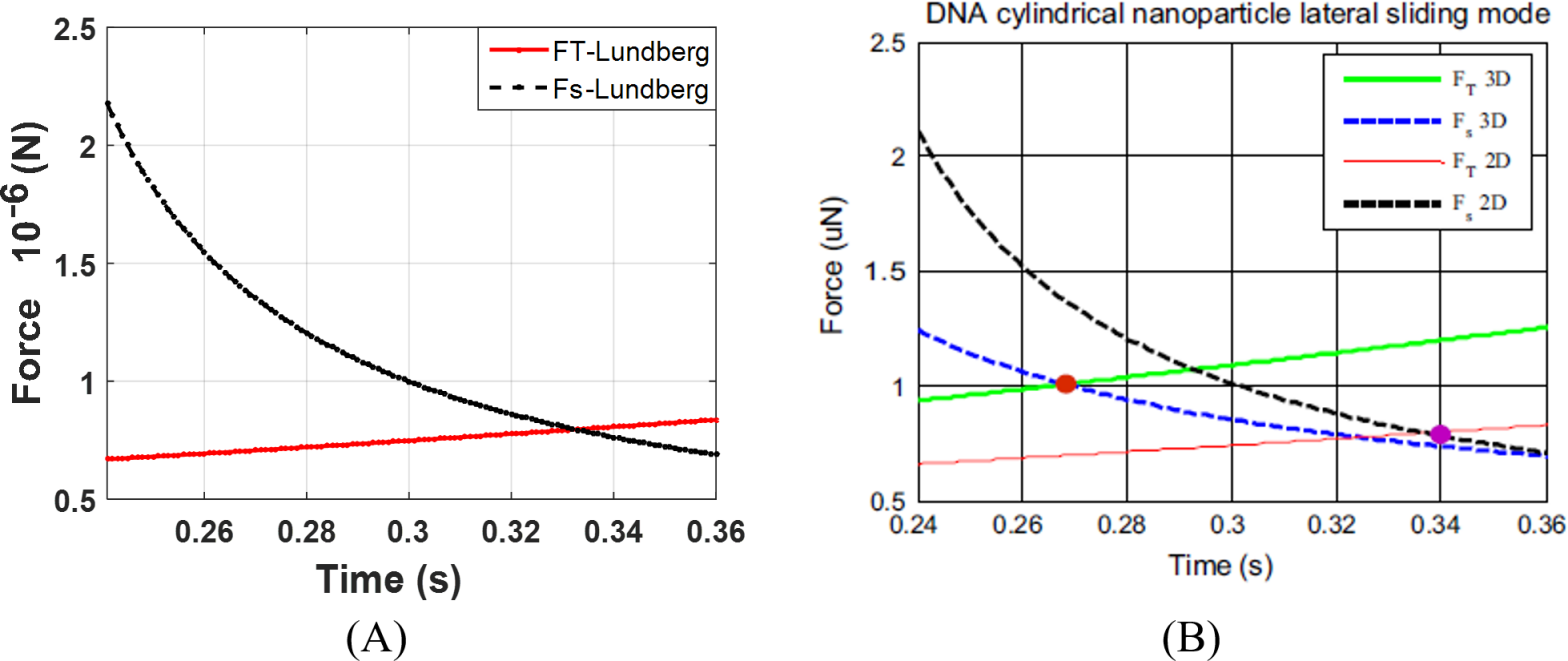
Comparison of the critical force and time simulation using the Lundberg cylindrical contact model for DNA. A) Present study simulation method. B) Korayem and Saraee simulation [16].

Since the validity of the method is mainly dependent on force simulations, the comparison of simulations of critical forces and times with other studies are presented in Figure 18 and Figure 19 by employing contact mechanics models that are in compliance with other studies.

Moradi et al. [8] studied the manipulation dynamics of a cylindrical nanorod made from polystyrene using a classical theory of continuum mechanics. In this section, by considering the information employed by Moradi et al. (Table 10), the deflections of a polystyrene nanorod are presented by including the size effects, followed by a comparison between the results.

**Table 10:** Employed information by Moradi et al. [8].

Critical force (nN)	Critical time (s)	Nanorod diameter (nm)	Nanorod length (nm)

18.8	0.18	85	1000

**Figure 20:**
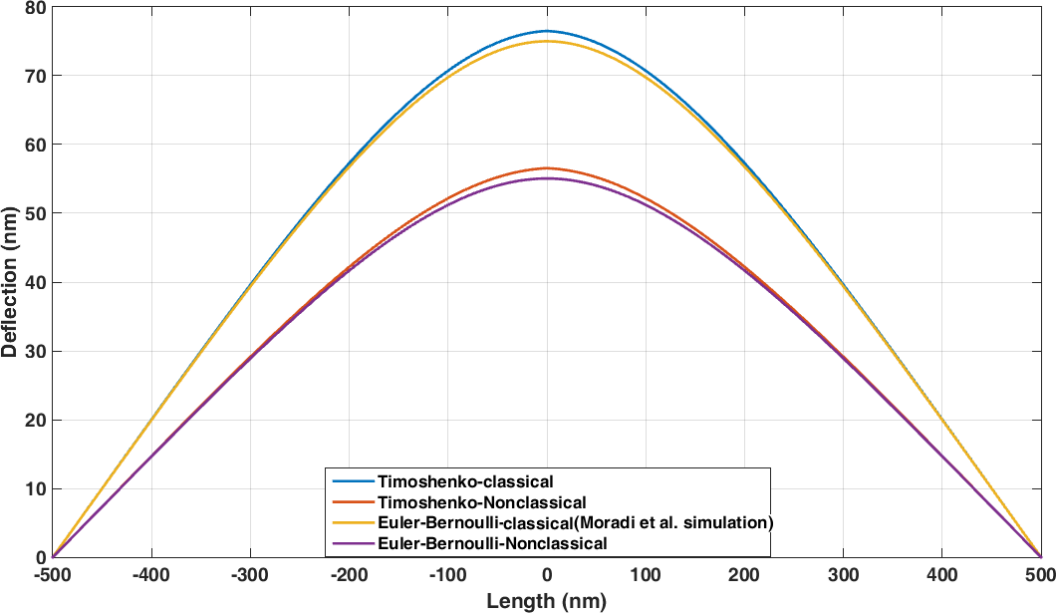
Comparison of the deflections of a polystyrene nanorod using classical and nonclassical models assuming *l*/*d* = 0.25.

Figure 20 and Table 11 show the deflections of the polystyrene nanorod according to different models with the loading conditions presented in the study of Moradi et al.; the results are obtained by considering *l*/*d* = 0.25. As observed, nonclassical models predict 25% lower maximum deformation compared with the classical Euler–Bernoulli beam model as in the study of Moradi et al.

**Table 11:** Comparison of the deflections of a polystyrene nanorod using classical and nonclassical models assuming *l*/*d* = 0.25.

Beam model	Maximum deflection (nm)	Difference from classical Timoshenko

classical Timoshenko	76.08	0
classical Euler–Bernoulli	74.60	1.95%
nonclassical Timoshenko	56.24	26.08%
nonclassical Euler–Bernoulli	54.76	28.02%

**Figure 21:**
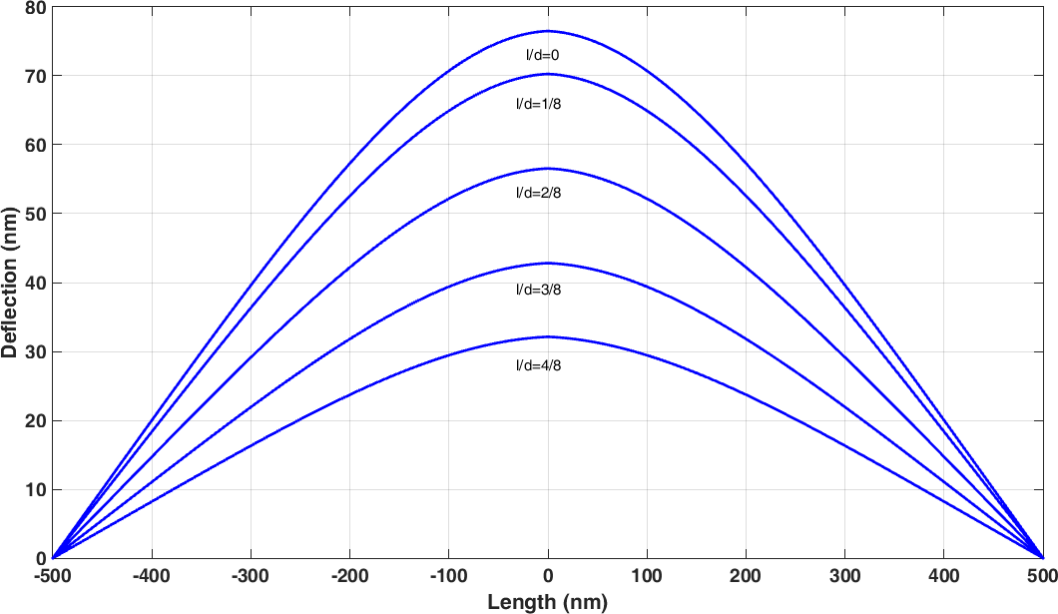
Comparison of polystyrene nanorod deflections in the nonclassical Timoshenko beam model with respect to *l*/*d*.

**Figure 22:**
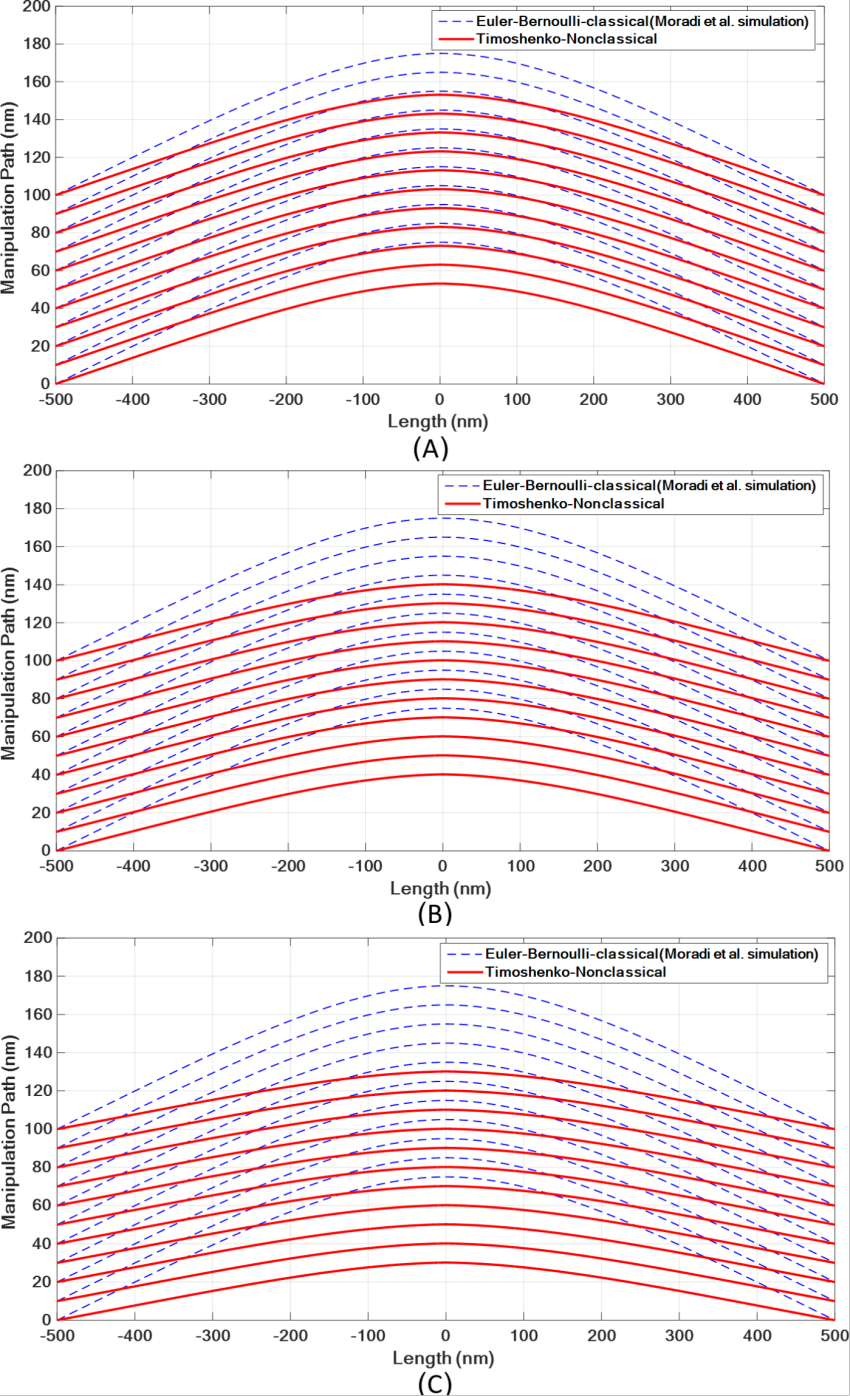
Simulation of the manipulation process of a polystyrene nanorod and comparison with the presented model by Moradi et al. [8]. A: *l*/*d* = 2/8. B: *l*/*d* = 3/8. C: *l*/*d* = 4/8.

By varying *l*/*d*, the deflections predicted in the nonclassical model change. Figure 21 and Table 12 show the deflections of the polystyrene nanorod based on the nonclassical Timoshenko model with respect to *l*/*d*. By increasing the value of *l*/*d*, lower deflections are obtained in nonclassical models. After obtaining the deflections, Moradi et al. carried out a 100 nm simulation on the polystyrene nanorod motion. The comparison of the results of the classical simulation with those of the nonclassical simulation shows lower deflections before the onset of motion and more required time for approaching target point (Figure 22 and Table 13) in nonclassical models.

**Table 12:** Maximum deflection of a polystyrene nanorod in the nonclassical Timoshenko beam model.

Material length scale parameter	*l*/*d* = 4/8	*l*/*d* = 3/8	*l*/*d* = 2/8	*l*/*d* = 1/8	*l* = 0

maximum deflection (nm)	31.93	42.58	56.24	69.89	76.08
difference from classical model	58.03%	44.06%	26.08%	8.11%	–

**Table 13:** Final position of the polystyrene nanorod using a nonclassical Timoshenko beam model.

Material length scale parameter	*l*/*d* = 4/8	*l*/*d* = 3/8	*l*/*d* = 2/8	*l*/*d* = 1/8	*l* = 0

final position (nm)	130.19	140.25	153.16	166.06	171.92
difference from classical model	24.27%	18.42%	10.91%	3.41%	–

**Figure 23:**
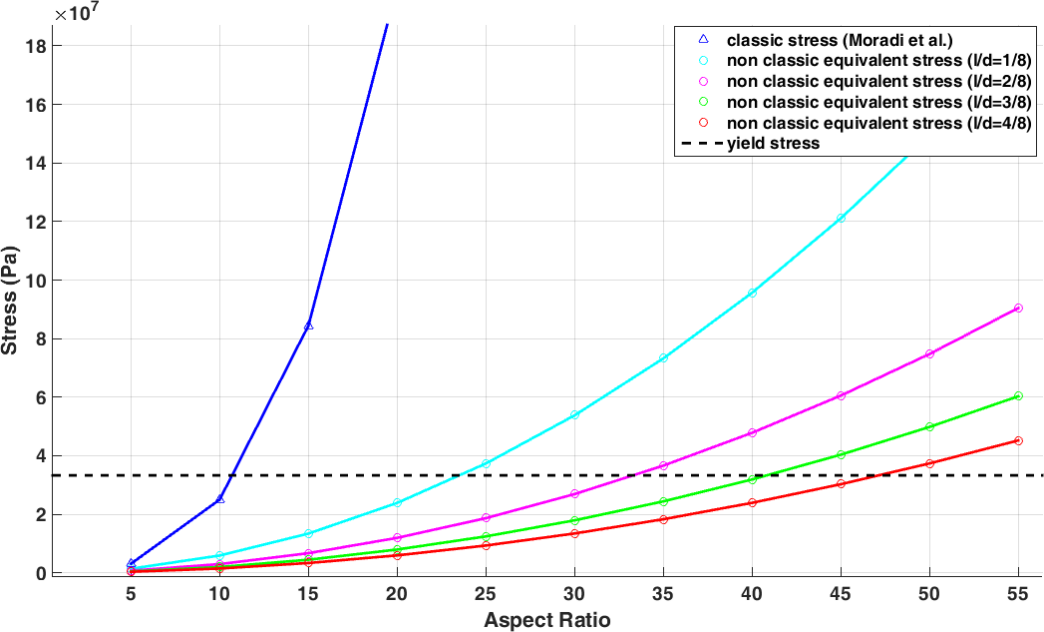
Variation of the failure aspect ratio versus *l*/*d* for a polystyrene nanorod.

As the results show, the difference between the classical and nonclassical models increases with increasing material length scale parameter. At *l*/*d* = 0.5, a difference of more than 24% is observed. In the study carried out by Moradi et al., the aspect ratio of 11.76 was calculated as the critical failure aspect ratio [33]. Figure 23 shows that the nonclassical theory predicts a higher aspect ratio. As observed in Table 14, the difference between the classical and nonclassical models could be more than 75% at *l*/*d* = 0.5.

**Table 14:** Variation of the failure aspect ratio versus *l*/*d* for a polystyrene nanorod.

Material length scale parameter	*l*/*d* = 4/8	*l*/*d* = 3/8	*l*/*d* = 2/8	*l*/*d* = 1/8	*l* = 0

failure aspect ratio	47.12	40.80	33.28	23.50	11.76
difference from classical model	75.08%	71.17%	64.66%	49.96%	–

## Conclusion

In this article, in order to fill the gap of previous studies which mainly focused on molecular dynamics and classical continuum mechanics models, dynamic and mechanical modeling of manipulation process were carried out using nonclassical continuum mechanics theories. Because the material length scale parameter was used in the equations and the symmetric couple stress tensor, MCST was selected for modeling purposes. First, the required critical forces and times for the onset of motion in each motion mode were simulated and the sliding on the substrate was found to be the dominant mode. In addition, deflections of the cylindrical gold nanoparticle in classical and nonclassical models were studied. The maximum predicted deflection by the nonclassical approach was 90% less than that of the classical one. Also, with a decreasing aspect ratio, the deflections decreased in both the classical and nonclassical models. However, the increment rate of nonclassical models was lower, and by increasing the aspect ratio, the difference between classical and nonclassical models became greater. Moreover, the sensitivity of the nonclassical model to the size dependence was studied, demonstrating that with an increase in the material length scale parameter, the deflections of nonclassical models decreased, while the difference with respect to classical models increased. To investigate the effect of nonclassical modeling in the manipulation dynamics of cylindrical nanoparticles, a gold nanoparticle with a length of 25 µm and aspect ratio of 30 was manipulated on a substrate by 200 nm at a speed of 50 nm/s.

The results showed that by increasing the aspect ratio, the difference between the classical and nonclassical models regarding the prediction of final position increased and reached the considerable value of 25% for the aspect ratio of 35. In addition, by decreasing the manipulation distance, the difference between the two models increased. In order to ensure the studied samples will not fail, classical and equivalent nonclassical stresses were calculated and the stress sensitivity to the change in aspect ratio was investigated, showing a notable difference between predictions using classical and nonclassical models (more than 212%). In the end, the results obtained from the dynamic simulation of a cylindrical polystyrene nanorod with a length of 1 µm simulated by the classical model were compared with those obtained from the nonclassical simulation. The findings showed that the effect of size is indeed significant. For instance, the difference between the classical and nonclassical models was more than 58% at *l*/*d* = 0.5, and the classical models cannot accurately predict this. In addition, from a nonclassical study of stresses, it appeared that the aspect ratio of 11.76 obtained by another researcher in the classical model does not provide a precise prediction of the failure aspect ratio, i.e., the obtained aspect ratio was overdesigned. This aspect ratio increased by 75% in nonclassical models and reached 47.12. The importance of this issue will be more relevant when the increment of the aspect ratio leads to small range deflections.
